# Community-based multisensory environments as preventive public health interventions for mental well-being in older adults: evidence from a large-scale study in China

**DOI:** 10.3389/fpubh.2025.1718222

**Published:** 2026-01-14

**Authors:** Han Zhang, Siqi Zhu

**Affiliations:** Intelligent Design Laboratory, Platform at the Intersection of Art and Technology, Central China Normal University, Wuhan, China

**Keywords:** community-based interventions, healthy aging, non-pharmacological interventions, public mental health, social participation

## Abstract

The accelerating demographic transition has brought the mental health of older adults to the forefront of public health concerns. Middle-aged and older adults (aged 50–69 years), often described as the “young-old,” are particularly vulnerable to early-stage psychological distress as they navigate shifts in social roles and community participation. Traditional approaches such as pharmacological treatment, counseling, and organized activities provide partial relief but lack personalization, scalability, and sustainable engagement. This study examined how immersive multisensory therapeutic spaces influence mental health among 1,897 community-dwelling adults in China. Participants experienced community-based immersive environments before completing a structured survey. Structural equation modeling showed that social participation significantly reduced psychological distress, which in turn increased acceptance of immersive environments. Acceptance further exerted a significant positive effect on mental health improvement, highlighting its role as a proximal mechanism of change. Cluster analyses identified distinct psychosocial profiles and sensory preferences, offering insights into subgroup-specific intervention strategies. These findings provide large-scale empirical evidence on the behavioral and psychosocial pathways through which multisensory environments support mental health in later life. Social participation and acceptance jointly emerged as critical drivers, suggesting that community-based immersive spaces can function as scalable, person-centered, and culturally relevant non-pharmacological interventions that complement existing public mental health strategies.

## 1 Introduction

The accelerating demographic shift toward population aging has placed the mental health of older adults at the center of public health agendas (1, 112). Middle-aged and older adults frequently experience psychological challenges such as loneliness, depression, and anxiety, which not only diminish quality of life but also impose sustained burdens on healthcare and welfare systems (2, 3). Traditional interventions, including pharmacological treatment (4), psychological counseling (5), and organized social activities (6) provide partial relief, their long-term sustainability and personalization remain limited, especially in the absence of integration with emerging digital health solutions (7). Therefore, there is an urgent need for innovative, technology-supported mental health strategies that can enhance emotional regulation, foster social participation, and be implemented sustain-ably in community contexts (8, 9).

Immersive multisensory environments have recently attracted attention as promising psychosocial interventions, integrating spatial design, interactive media, and affective computing into coherent therapeutic settings (10). Originally applied in artistic and entertainment contexts (11), these environments are increasingly adapted for health promotion through panoramic projection, spatialized sound, interactive lighting, and tactile feedback (12–14). By simultaneously engaging visual, auditory, and tactile modalities, they have the potential to enhance emotional regulation, reduce psychological distress, and foster social connectedness among older adults (15–18). Compared with virtual reality (VR) or mixed reality (MR) systems, which may present cognitive and ergonomic challenges, community-based immersive spaces provide tangible, socially shared, and accessible experiences that are more suitable for older populations (18–20).

The “young-old” demographic, typically defined as adults aged 50–69 years, represents a transitional group navigating retirement and shifts in social roles, making them particularly vulnerable to psychological distress (21). Experiences of loneliness, identity change, and diminished social networks are common during this stage of life (22, 23). Nevertheless, this group often shows relatively high adaptability to digital technologies (24), making them an appropriate population for evaluating community-based multisensory interventions that integrate participatory and therapeutic components. Understanding how young-old adults perceive and engage with auditory, visual, and interactive modules, and how these relate to emotional regulation, can provide essential insights for tailoring preventive mental health strategies (25).

Despite increasing interest in immersive interventions, empirical evidence regarding their psychological mechanisms remains limited. Systematic reviews suggest that virtual reality and multisensory programs can reduce stress and improve mood among older adults (26–28). However, few studies have examined the differential contributions of specific sensory modalities such as visual stimuli, auditory ambience, and tactile feedback to psychological outcomes. In addition, the functional needs and preferences of older adults regarding sensory configurations remain underexplored (29–31). Importantly, the application of immersive technologies in age-friendly, community-based contexts has received little empirical validation, particularly for populations facing digital literacy and accessibility challenges (32).

Recent developments in digital therapeutics (DTx) and ubiquitous health (uHealth) platforms highlight the importance of incorporating multimodal interaction into scalable public mental health interventions (33). Research on smart environments, wearable-assisted interventions, and sensor-driven biofeedback suggests that multisensory engagement can enhance adherence, personalization, and long-term effectiveness in digital mental health programs (34). Nevertheless, the translation of such innovations into accessible, community-based formats for older adults remains insufficiently studied, particularly given potential barriers related to digital literacy and accessibility (35).

To address these gaps, the present study proposes a conceptual framework linking multisensory spatial design, multimodal interaction, social participation, and mental health outcomes in older adults. Drawing on a large-scale dataset of 1,897 participants in China, we employed structural equation modeling and clustering analysis to evaluate the overall effectiveness of community-based immersive therapeutic spaces, examine the differential impact of distinct sensory modules, and identify functional needs and preferences. This study situates immersive therapeutic environments within the broader domain of public mental health, conceptualizing them not merely as design innovations but as psychosocial interventions that combine digital media, behavioral science, and evidence-based strategies to promote sustainable mental health benefits for aging populations.

## 2 Related work

### 2.1 Digital and multisensory interventions in public health

The global trend of population aging has intensified the prevalence of mental health problems such as depression and anxiety among older adults, with global rates ranging from 31.7% to over 40% in developing countries (36–38). Existing interventions such as pharmacological treatments (39), social engagement programs (40), peer-based support (41), and mindfulness-based therapies (42) provide partial relief but are limited by issues of long-term sustainability, personalization, and integration with digital technologies (5, 43). Pharmacological strategies, although effective for some, often suffer from adherence problems and side effects (44, 113).

Recent advances in DTx and uHealth platforms demonstrate that technology can extend care beyond clinical settings and provide scalable support for mental health (33). These systems utilize multimodal interaction technologies, including visual, auditory, and haptic feedback, to provide personalized and scalable therapeutic interventions. Meta-analyses of internet-based cognitive behavioral therapy (iCBT) confirm significant reductions in depressive symptoms among older adults, especially when interventions are multimodal and socially supported (45). Similarly, mindfulness-based digital interventions show improvements in emotion regulation and resilience (42). Virtual reality (VR) and other immersive technologies further enhance affective engagement and treatment efficacy, highlighting their potential as complementary psychosocial interventions (46, 47).

Nevertheless, adoption of digital health tools by older adults remains inconsistent. Evidence indicates that barriers such as usability, trust, and digital literacy substantially limit sustained engagement (48). Although emerging approaches, such as voice-assisted systems and AR-based training, aim to improve accessibility and provide more natural interfaces (49, 50), challenges of long-term adherence remain. These observations suggest that multimodal design should be conceptualized not merely as a technical feature but as a determinant of therapeutic effectiveness, user acceptance, and sustainability in mental health care (33, 46, 47). Extending these principles into community-based physical immersive spaces therefore represents a promising pathway for developing scalable psychosocial interventions that address both individual emotional regulation and broader public health goals.

### 2.2 Immersive multisensory environments in community health interventions

Immersive environments have long drawn on sound and light technologies to enhance sensory engagement, with early applications traceable to theatrical and artistic practices in the eighteenth century (51–53). While installation art in the late twentieth century further advanced spatial immersion through interactive media and large-scale exhibitions (54–56), these approaches primarily highlighted the experiential and psychological effects of sensory stimulation rather than their implications for health.

In recent decades, immersive environments have been increasingly translated into functional applications across public health, education, and eldercare. For example, community-based installations in museums, senior centers, and health facilities have been shown to stimulate social interaction, reduce stress, and enhance subjective well-being (57, 58). Evidence from digital health research further indicates that immersive environments using VR, AR, and projection-based systems can improve emotional regulation, increase presence, and support cognitive engagement, thereby demonstrating clear psychosocial benefits (46, 47, 59).

The integration of emerging technologies such as artificial intelligence, biosensors, and multimodal data analytics has broadened the potential of these interventions. AI-enabled emotion recognition and adaptive content delivery allow therapeutic spaces to respond dynamically to users’ affective states, while multimodal sensor systems enable personalized feedback and continuous monitoring of behavioral outcomes (60, 61). This convergence illustrates a transition from aesthetic experimentation toward evidence-based psychosocial interventions that are relevant to public health.

Compared with VR or MR headsets, which may present ergonomic and cognitive barriers for older adults, community-based physical immersive spaces enhanced with multimodal technologies offer low-barrier, tangible, and socially shared experiences. This makes them particularly well suited for public health interventions targeting older populations (62–64). Empirical studies confirm that such environments can foster social participation, strengthen emotional connectedness, and reduce depressive symptoms among older adults (57, 65, 66). Taken together, these developments underscore the growing recognition of immersive multisensory environments as a viable framework for sustainable, community-based interventions within behavioral and public health sciences.

### 2.3 Multisensory stimulation and psychosocial outcomes in older adults

Sensory stimulation has long been recognized as a foundation for emotional regulation and psychological well-being. Evidence shows that auditory interventions such as music therapy effectively reduce stress and anxiety (67), visual exposure to natural scenes alleviates tension and enhances positive affect (68), tactile stimulation fosters comfort and relaxation (69), and olfactory cues are associated with lower depressive symptoms (70). Public health studies increasingly suggest that integrating these modalities within multisensory programs provides greater benefits than unimodal approaches, with improvements reported in mood regulation, cognitive performance, and social participation among older adults (71, 114).

Recent research has further demonstrated that multisensory environments tailored for older populations can reduce depressive symptoms and enhance resilience. Interventions that combine auditory ambiences, soothing visual imagery, and tactile engagement have produced measurable reductions in anxiety and depression scores, while also improving subjective well-being (72–75). Advances in multimodal biofeedback allow real-time adjustment of sensory inputs, reinforcing personalization and supporting emotion regulation processes central to behavioral science models of stress recovery and cognitive load management (76, 77).

Beyond individual emotional benefits, multisensory design plays a critical role in promoting social participation, which is a recognized determinant of health in aging populations. Shared interactive experiences strengthen social connectedness, reduce loneliness, and promote a sense of belonging (78, 79). Social interaction has consistently been linked with better psychological outcomes, including higher self-esteem and reduced depressive symptoms (80–82). Community-based interventions incorporating gamified and embodied multisensory activities have been shown to enhance older adults’ engagement, supporting social identity and aligning with public health models of behavioral activation in late-life mental health (50, 60).

Taken together, these findings indicate that multisensory interaction functions as a dual pathway for improving mental health: by directly supporting emotional regulation and by strengthening social integration. Nevertheless, limited empirical evidence exists regarding how specific sensory modalities, such as auditory ambience, visual scene design, or tactile engagement, align with older adults’ functional needs and preferences in community settings. Addressing this gap requires systematic, large-scale studies that situate multisensory environments within public health frameworks, which motivates the present study.

### 2.4 Research gaps and study objectives

Although previous research has demonstrated the potential of digital therapeutics, multisensory interventions, and immersive environments for supporting mental health in older adults, several critical limitations remain. First, much of the existing evidence is derived from small-scale or short-term studies, which restricts generalizability and limits conclusions about sustainability in real-world settings. Second, prior work has predominantly focused on laboratory-based or headset-driven VR/AR applications, while relatively little attention has been given to community-based immersive spaces that integrate multimodal interaction in age-friendly and socially inclusive environments. Third, the specific functional needs and sensory preferences of older adults, including how auditory, visual, and tactile modules contribute to emotion regulation, have not been systematically investigated. Finally, there is a lack of comprehensive models that connect multisensory spatial design, social participation, and psychological outcomes using large-scale datasets and rigorous statistical methods.

To address these gaps, the present study develops a conceptual framework linking multisensory spatial design, multimodal interaction features, social participation, and mental health outcomes in older adults. Using a large-scale dataset of 1,897 community-dwelling adults aged 50–69 years in China, we apply structural equation modeling (SEM) and clustering analysis to: (1) evaluate the overall effectiveness of community-based immersive therapeutic spaces; (2) identify the differential impacts of specific sensory modules; (3) examine the mediating and moderating roles of social participation and emotional regulation; and (4) provide evidence for scalable, sustainable, and culturally relevant non-pharmacological interventions in aging populations. By situating immersive environments within a public health framework, this study aims to advance understanding of preventive and community-based strategies for promoting psychological well-being among older adults.

## 3 Materials and methods

### 3.1 Conceptual framework

Immersive multisensory environments are defined in this study as community-based spaces that integrate visual, auditory, and interactive features to promote psychological well-being among older adults. Previous research indicates that such environments can reduce depression, anxiety, and stress by facilitating emotional regulation and stress recovery (83–85). On this basis, immersive environments are expected to exert direct positive effects on mental health outcomes.

Beyond direct pathways, social participation is widely recognized as a key determinant of psychological well-being. Engagement in community and social activities enhances emotional connectedness, reduces loneliness, and fosters a sense of belonging (86–89). In this study, social participation is conceptualized as a mediating mechanism that explains how exposure to multisensory environments translates into improved mental health outcomes.

Intervention effectiveness may further depend on individual baseline conditions. Evidence suggests that individuals with higher levels of psychological distress may experience weaker restorative benefits, while those with stronger social resources benefit more from community-based interventions (69, 90). Thus, baseline psychological distress is hypothesized to moderate the impact of multisensory interventions on mental health.

In addition, different sensory modalities are expected to exert differentiated impacts. Visual exposure to natural scenes has been associated with stress reduction (91–94), auditory ambience such as music alleviates depression and anxiety (95–97), and interactive features enhance engagement and emotional resonance (98, 99). It is important to assess the differentiated effects of sensory modalities.

**Figure 1:**
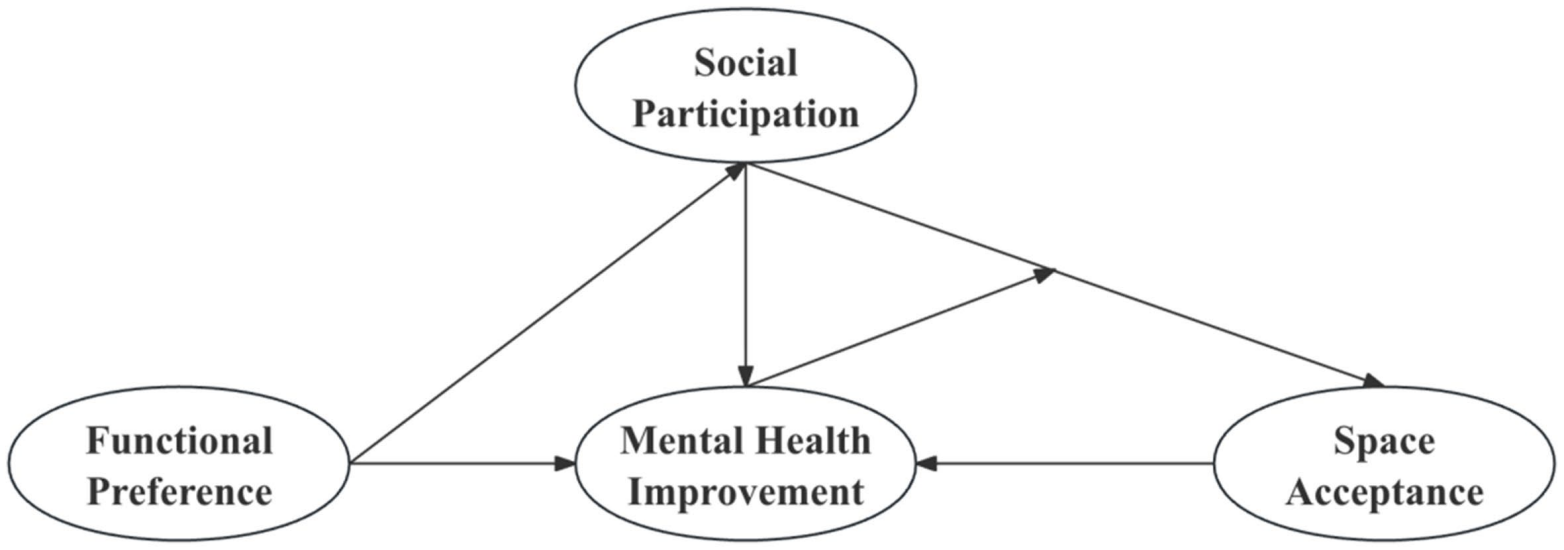
Design and implementation of community-based immersive therapeutic space.

This study proposes a conceptual framework linking multisensory environments, social participation, and psychological outcomes, while accounting for moderating and differentiated effects. Based on this framework, the following hypotheses are tested (Figure 1):

*H1*: Immersive multisensory environments have a significant positive association with mental health improvement among older adults.

*H2*: Social participation mediates the association between immersive multisensory environments and mental health improvement.

*H3*: Baseline psychological distress moderates the association between immersive multisensory interventions and mental health improvement.

*H4*: Different sensory modalities (visual, auditory, interactive) show distinct associations with mental health improvement.

### 3.2 Study design

**Figure 2:**
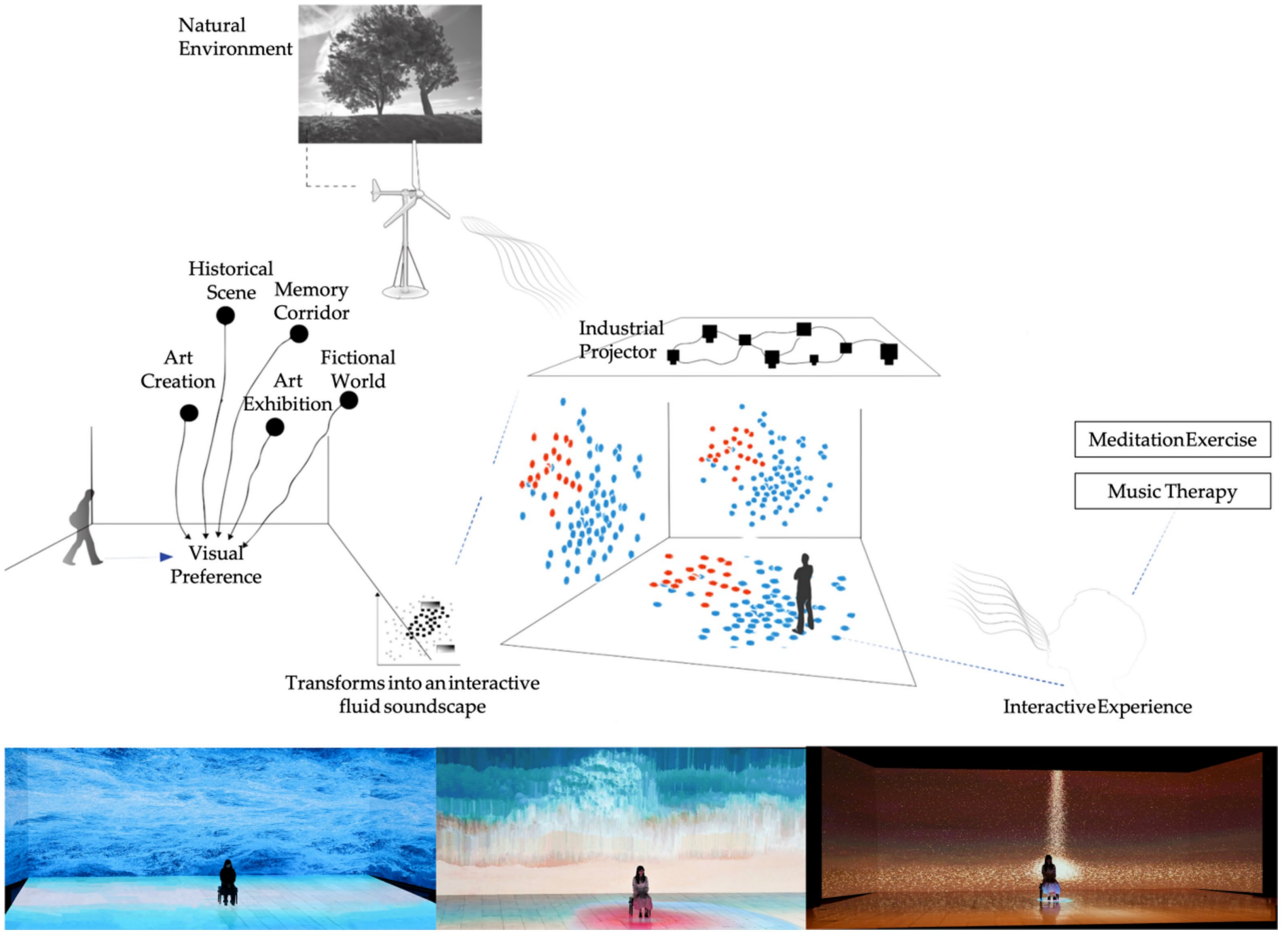
Design and implementation of community-based immersive therapeutic space.

This study employed a cross-sectional survey design to investigate the impact of immersive multisensory environments on mental health among community-dwelling older adults. The target population was adults aged 50–69 years, often referred to as the “young-old,” who are at risk of psychological distress during life transitions but remain receptive to preventive interventions. To enhance ecological validity, participants were invited to visit and directly experience a community-based immersive therapeutic space prior to survey completion (Figure 2). The study framework was structured to test direct, mediating, and moderating pathways linking multisensory exposure, social participation, and mental health outcomes.

### 3.3 Questionnaire design

This study employed a structured questionnaire to measure the key constructs in the conceptual framework, which included social participation, attitudes toward non-pharmacological interventions, baseline mental health status, and functional preferences for multisensory environments. Each construct was selected based on prior evidence linking it to late-life psychological well-being and community health. The questionnaire was divided into five sections:

(1) Basic Information: Collected demographic data including gender, age, education, marital status, income sources, and living arrangements.
(2) Social Relationships and Social Participation: Assessed social connectedness, frequency of community engagement, and participation in social activities, drawing on validated measures from public health and social epidemiology (86, 88, 89).
(3) Attitudes Toward Non-Pharmacological Interventions: Measured participants’ openness to, and acceptance of, alternative psychosocial interventions, including community-based immersive environments.
(4) Baseline Mental Health Status: Measured using the Simplified Chinese version of the Depression, Anxiety, and Stress Scale (DASS-21) (100). Previous studies have demonstrated the reliability and validity of the DASS-21 among Chinese older adult populations, supporting its suitability for the present research context.
(5) Functional Preferences: Captured participants’ preferences for specific sensory modalities (e.g., visual, auditory, interactive features) and their perceived effectiveness in promoting psychological well-being.

To minimize comprehension difficulties and ensure cultural appropriateness, participants were provided with standardized textual explanations and visual aids illustrating the immersive therapeutic spaces, rather than extended demonstrations. A pilot test with 35 participants was conducted to refine item wording and ensure clarity. The study protocol was approved by the institutional ethics committee. All participants provided written informed consent, and data collection adhered to principles of confidentiality, voluntary participation, and the right to withdraw at any stage.

### 3.4 Data collection

Data collection was conducted between May and June 2024 in urban and suburban community centers in China. Trained research assistants provided standardized instructions, guided participants through the immersive experience, and offered clarification during questionnaire completion. Surveys were administered in a face-to-face format to minimize comprehension difficulties common in older populations. A total of 1,900 questionnaires were distributed, of which 1,897 were valid, yielding a completion rate of 99.8%. The sample was balanced by gender (959 men and 938 women) and showed diversity in education and socioeconomic status. The final sample size exceeded recommended thresholds for structural equation modeling (SEM), ensuring sufficient statistical power for hypothesis testing and subgroup analyses.

### 3.5 Reliability and validity assessment

Internal consistency was evaluated using Cronbach’s alpha. The social participation scale yielded 0.938, the DASS-21 total scale 0.960 (subscales: depression 0.962, anxiety 0.952, stress 0.964), and the attitudes toward non-pharmacological interventions scale 0.605. These results indicated high reliability for most constructs and moderate reliability for the latter.

Construct validity was confirmed via exploratory factor analysis (EFA) and confirmatory factor analysis (CFA). KMO values exceeded 0.90 for all constructs, and Bartlett’s test of sphericity was significant (*p* < 0.001). Variance explained exceeded 80%, with factor loadings > 0.5. CFA results showed standardized loadings > 0.6, AVE > 0.5, and discriminant validity, as the square root of AVE exceeded inter-construct correlations.

### 3.6 Data analysis

Statistical analyses were conducted with SPSS 26.0 and AMOS 21.0. Descriptive statistics summarized demographic data and variable distributions. Hypotheses were tested using structural equation modeling (SEM). Mediation was examined via bootstrap resampling (5,000 iterations), while moderation was assessed through simple slope analysis. Model fit was evaluated using RMSEA, CFI, TLI, and SRMR. In addition, *k*-means clustering analysis was conducted to identify participant subgroups based on functional preferences and engagement patterns with multisensory modules. This approach provided further insights into heterogeneity in psychosocial responses and informed the identification of tailored intervention strategies.

## 4 Results

### 4.1 Descriptive statistics and sample characteristics

A total of 1,897 valid responses were collected from community-dwelling middle-aged and older adults aged 50 to 69 years. The gender distribution was balanced, with 50.6% male and 49.4% female participants. Most respondents were aged 60–69 years (66.3%). Regarding education, 40.7% had completed middle school, 32.3% had a vocational or high school degree, and 4.6% held a bachelor’s degree or higher.

The majority of participants were married (92.3%). Self-reported health conditions were rated as good by 16.4%, average by 68.4%, and poor by 15.1%. In terms of income, 36.1% reported ≤1,000 RMB per month, while 38.2% earned 1,001–2,000 RMB. Expenditure was balanced for 65.3% of respondents, with 17.3% reporting surplus and 17.4% reporting difficulty maintaining expenditure.

With respect to living arrangements, 47.7% lived with their children, 39.2% with a spouse, 7.4% alone, and 5.7% in a nursing home. Nearly all participants (98.7%) had children. Regarding older adults care preferences, 39.9% preferred family-based care, 38.9% institutional care, and 21.3% community-based care.

A total of 62.8% of respondents agreed or strongly agreed that non-pharmacological interventions, such as art-based activities, could improve mental health. Detailed demographic information is presented in Table 1.

**Table 1:** Basic information.

Variable	Category	Proportion/% (*N* = 1897)
Gender	Male	50.6
	Female	49.4
Age	50–59	33.7
	60–69	66.3
Education level	Primary school or below	15.0
	Middle school	40.7
	Vocational/High school	32.3
	Associate degree	7.4
	Bachelor’s degree	4.2
	Master’s degree or above	0.4
Marital status	Married	92.3
	Divorced	4.0
	Widowed	3.7
Health condition	Good	16.4
	Average	68.4
	Poor	15.1
Current living situation	Living with children	47.7
	Living with spouse	39.2
	Living alone	7.4
	In a nursing home	5.7
Children status	Has children	98.7
	No children	1.3
Preferred older adults care option	Family-based care	39.9
	Institutional care	38.9
	Community-based care	21.3
Income source	Pension	25.0
	Savings	25.9
	Spouse’s support	22.5
	Children’s support	26.1
	Government aid	0.4
Income	≤1,000 CNY	36.1
	1,001–2000 CNY	38.2
	2001–5,000 CNY	11.6
	≥5,001 CNY	14.1
Spending condition	Surplus	17.3
	Balanced	65.3
	Difficult to maintain	17.4
Do you believe art healing can help improve mental health	Strongly agree	44.2
	Agree	18.6
	Neutral	19.7
	Disagree	13.1
	Strongly disagree	4.5

### 4.2 Mediation analysis

The mediating role of mental health in the relationship between social participation and the acceptance of immersive therapeutic spaces was tested using SEM with AMOS 21.0. The model demonstrated an excellent fit to the data (*χ*^2^/df = 4.946, RMSEA = 0.046, NFI = 0.966, RFI = 0.963, IFI = 0.973, TLI = 0.970, CFI = 0.973, GFI = 0.935). Table 2 presents the path coefficients, and Table 3 summarizes the indirect effects.

**Table 2:** A test of the relationship between social engagement, mental health, and the impact of immersive healing space acceptance variables.

Relationship			*b*	*β*	SE	*t*	*p*
Social participation	→	Depression	−0.299	−0.335	0.021	−14.185	<0.001
Social participation	→	Anxiety	−0.242	−0.324	0.018	−13.533	<0.001
Social participation	→	Stress	−0.346	−0.399	0.020	−17.014	<0.001
Depression	→	Acceptance of immersive therapeutic space	−0.152	−0.191	0.021	−7.391	<0.001
Anxiety		Acceptance of immersive therapeutic space	−0.163	−0.172	0.024	−6.641	<0.001
Stress	→	Acceptance of immersive therapeutic space	−0.136	−0.167	0.022	−6.289	<0.001
Social participation	→	Acceptance of immersive therapeutic space	0.156	0.221	0.021	7.348	<0.001

**Table 3:** A test of the mediating effect of social engagement, mental health, and acceptance of immersive healing spaces.

Mediated pathway	Effect size	95% lower	95% upper	SE
Social participation →Depression → Acceptance of immersive therapeutic space	0.045	0.030	0.062	0.008
Social participation →Anxiety → Acceptance of immersive therapeutic space	0.039	0.025	0.057	0.008
Social participation →Stress → Acceptance of immersive therapeutic space	0.047	0.028	0.067	0.010

Path analysis showed that social participation had significant negative effects on depression (*β* = −0.335, *p* < 0.001), anxiety (*β* = −0.324, *p* < 0.001), and stress (*β* = −0.399, *p* < 0.001). In turn, all three indicators negatively predicted space acceptance (depression: *β* = −0.191, *p* < 0.001; anxiety: *β* = −0.172, *p* < 0.001; stress: *β* = −0.167, *p* < 0.001). Social participation also exerted a significant direct positive effect on space acceptance (*β* = 0.221, *p* < 0.001).

Bootstrap analysis with 5,000 resamples confirmed the significance of the indirect effects. The mediating effects through depression, anxiety, and stress were 0.045, 0.039, and 0.047, respectively, with 95% confidence intervals excluding zero.

Taken together, these findings indicate that social participation reduces psychological distress, which in turn enhances openness to community-based immersive interventions. This sequential pathway highlights the importance of strengthening social resources as a means to improve both mental health and the acceptance of innovative public health interventions.

### 4.3 Moderation analysis

**Figure 3:**
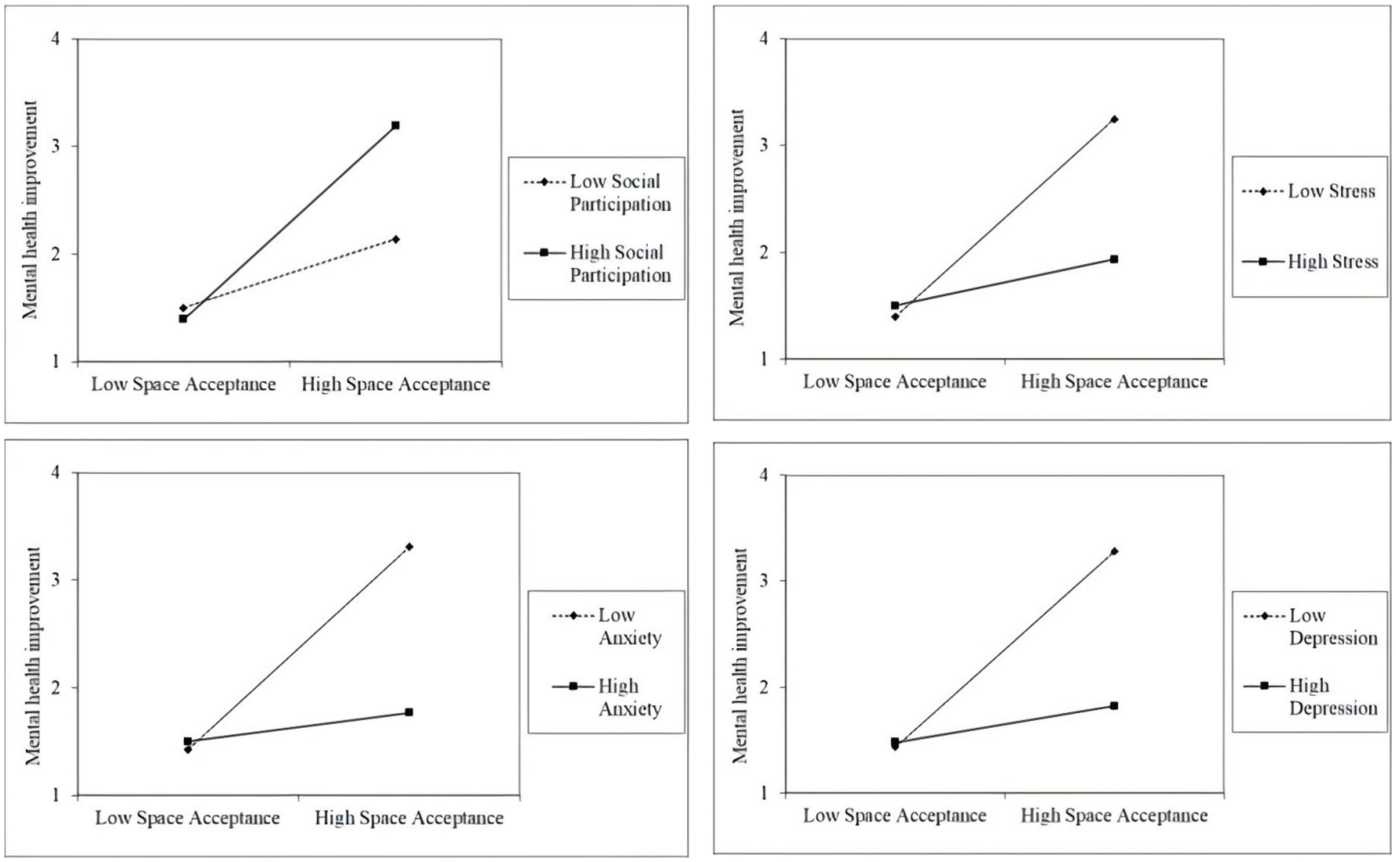
Simple slope of moderating effect (chart from left to right: social engagement, depression, anxiety, stress).

The moderating effects of social participation and baseline psychological distress were examined using hierarchical regression analyses. Acceptance of immersive therapeutic spaces was included as the predictor, while social participation and baseline mental health indicators (depression, anxiety, stress) were specified as moderators (Table 4 and Figure 3).

**Table 4:** An examination of the moderating effects of social engagement, a baseline mental health status variable, on the effectiveness of an immersive healing space intervention.

Dependent variable	Mental health improvement							
	Model 1	Model 2	Model 3	Model 4	Model 5	Model 6	Model 7	Model 8
Constant	2.152***	2.055***	2.152***	2.005***	2.152***	2.000***	2.152***	2.019***
Acceptance of immersive therapeutic space	0.654***	0.609***	0.624***	0.546***	0.625***	0.539***	0.629***	0.571***
Social participation	0.210***	0.237***						
Depression			−0.255***	−0.353***				
Anxiety					−0.262***	−0.367***		
Stress							−0.253***	−0.303***
Social participation × Acceptance of immersive space		0.291***						
Depression × Acceptance of immersive space				−0.376***				
Anxiety × Acceptance of immersive space						−0.404***		
Stress × Acceptance of immersive space								−0.355***
*R* ^2^	0.365	0.413	0.375	0.445	0.378	0.459	0.375	0.443
Adjust *R*^2^	0.364	0.412	0.374	0.444	0.377	0.458	0.374	0.442
*F*	543.530	443.592	568.540	505.855	574.517	536.127	568.153	502.071
*p*	<0.001	<0.001	<0.001	<0.001	<0.001	<0.001	<0.001	<0.001

***Indicates statistical significance at *p* < 0.001.

Results showed that social participation significantly strengthened the positive association between acceptance and mental health improvement (*b* = 0.291, *p* < 0.001). In contrast, baseline psychological distress weakened the effectiveness of acceptance. Specifically, significant negative interaction effects were observed for depression × acceptance (*b* = −0.376, *p* < 0.001), anxiety × acceptance (*b* = −0.404, *p* < 0.001), and stress × acceptance (*b* = −0.355, *p* < 0.001).

Simple slope analyses provided further insights. Among individuals with lower levels of depression, anxiety, or stress, the positive association between acceptance and mental health improvement was markedly stronger compared with those reporting higher baseline distress.

These findings suggest that the effectiveness of immersive community-based interventions is conditioned by individuals’ initial psychological resources. While socially active individuals derive greater benefits, those with higher psychological distress may require additional guidance or supportive measures to achieve similar improvements. This highlights the need for tailoring community interventions to the psychosocial profiles of older adults in order to maximize their impact.

### 4.4 Differential effects of functional modules

To examine variations in functional preferences and their associations with mental health outcomes, a *K*-means clustering analysis was conducted. Using the Elbow Method, the optimal number of clusters was determined to be three, yielding cluster sizes of 630, 703 and 564 participants, respectively.

Significant differences across clusters were observed for all preference variables (*p* < 0.001). Group 1 demonstrated stronger preferences for art creation, art exhibitions, and meditation, coupled with higher adaptability to environmental changes. Group 2 was characterized by a preference for natural environments, fictional worlds, and memory corridors, but displayed lower adaptability. Group 3 prioritized interactive experiences, music therapy, and meditation, with additional interest in historical scenes and memory corridors. The results are summarized in Table 5.

**Table 5:** Final cluster centers and ANOVA results for functional and sensory preference variables.

Variables	Final cluster centers			*F*	*p*
	Group 1 (*n* = 630)	Group 1 (*n* = 703)	Group 1 (*n* = 564)		
Interactive experience	0	0	1	95.154	<0.001
Music therapy	0	0	1	72.962	<0.001
Art creation	1	0	0	59.977	<0.001
Art exhibition	1	0	0	100.777	<0.001
Meditation exercise	1	0	1	255.139	<0.001
Natural environment	0	1	0	218.999	<0.001
Historical scenes	0	0	1	158.533	<0.001
Fictional worlds	0	1	0	276.988	<0.001
Art gallery	1	0	0	88.474	<0.001
Memory corridor	0	1	1	110.0 14	<0.001
Environmental loyalty	2	1	4	2178.46	<0.001
Auditory preference	3	3	5	487.891	<0.001
Visual preference	3	1	2	1454.41	<0.001

To provide a psychosocial interpretation, clusters were compared on social participation, baseline psychological distress, and acceptance (Table 6). Group 2 exhibited the lowest levels of social participation and the highest levels of depression, anxiety, and stress, along with the weakest acceptance of immersive spaces. In contrast, Group 3 reported the highest levels of social participation, acceptance, and mental health improvement. Group 1 presented intermediate scores across most indicators.

**Table 6:** Comparisons of psychosocial characteristics across clusters (ANOVA results).

Variables	Group 1	Group 2	Group 3	*F*	*p*
Social participation	3.15 ± 1.26	2.99 ± 1.25	3.26 ± 1.25	7.287	0.001
Depression	1.99 ± 1.04	2.12 ± 1.06	1.88 ± 1.02	8.297	0.000
Anxiety	1.94 ± 0.93	2.04 ± 0.93	1.85 ± 0.91	7.020	0.001
Stress	2.17 ± 1.06	2.23 ± 1.07	2.02 ± 1.03	6.337	0.002
Space acceptance	2.10 ± 1.02	1.99 ± 1.00	2.25 ± 1.05	10.135	0.000
Art healing attitude	3.24 ± 1.13	3.12 ± 1.17	3.35 ± 1.12	6.473	0.002
Mental health improvement	2.19 ± 1.27	1.91 ± 1.06	2.41 ± 1.37	26.541	0.000

Regression analyses further clarified the role of individual functional modules (Table 7). Activity-based modules such as music therapy (*β* = 0.122, *p* < 0.001), art creation (*β* = 0.098, *p* < 0.001), and meditation (*β* = 0.077, *p* = 0.001) were significantly associated with better mental health outcomes. Spatial content preferences, including natural environments (*β* = 0.110, *p* < 0.001), historical scenes (*β* = 0.086, *p* < 0.001), fictional worlds (*β* = 0.070, *p* = 0.004), art galleries (*β* = 0.077, *p* = 0.001), and memory corridors (*β* = 0.058, *p* = 0.016), also showed positive associations. Among sensory modalities, auditory (*β* = 0.090, *p* < 0.001) and visual preferences (*β* = 0.057, *p* = 0.031) were significant predictors. The regression coefficients and model statistics are presented in Table 7.

**Table 7:** Regression analyses of functional modules predicting mental health improvement.

Variables	*b*	SE	*β*	*t*	*p*
Interactive experience	0.117	0.073	0.047	1.605	0.109
Music therapy	0.303	0.072	0.122	4.231	0.000
Art creation	0.242	0.064	0.098	3.786	0.000
Art exhibition	0.131	0.072	0.050	1.824	0.068
Meditation exercise	0.202	0.063	0.077	3.194	0.001
Natural environment	0.276	0.061	0.110	4.502	0.000
Historical scene	0.215	0.060	0.086	3.568	0.000
Fictional world	0.177	0.062	0.070	2.847	0.004
Art gallery	0.192	0.056	0.077	3.419	0.001
Memory corridor	0.145	0.060	0.058	2.411	0.016
Environmental loyalty	0.077	0.031	0.072	2.512	0.012
Auditory preference	0.110	0.030	0.090	3.618	0.000
Visual preference	0.070	0.032	0.057	2.158	0.310

To further contextualize the clusters from a socioeconomic perspective, post-hoc comparisons were conducted across key demographic variables, including income level, education, and living arrangements (see Supplementary Table S1). Chi-square tests indicated that cluster membership was not significantly associated with income [*χ*^2^(6) = 2.32, *p* = 0.888, Cramer’s *V* = 0.025], education level [*χ*^2^(10) = 14.86, *p* = 0.137, Cramer’s *V* = 0.063], or living arrangement [*χ*^2^(6) = 4.62, *p* = 0.594, Cramer’s *V* = 0.035]. These results suggest that the identified clusters primarily reflect differences in functional preferences and psychosocial profiles rather than being driven by basic socioeconomic composition, although minor distributional trends (e.g., a slightly higher proportion of lower education in Group 2) were observed.

Overall, these findings highlight that activity-based modules exhibited stronger and more consistent positive associations with mental health improvement compared with purely visual content. This aligns with behavioral science perspectives suggesting that active engagement promotes both emotional regulation and social connectedness. From a public health standpoint, tailoring interventions to subgroup preferences may improve both acceptance and therapeutic efficacy, particularly for socially vulnerable older adults.

### 4.5 Complete pathway analysis

To comprehensively examine the mechanisms through which immersive therapeutic spaces influence mental health outcomes, a full structural equation model was estimated, incorporating social participation, attitudes toward non-pharmacological interventions, acceptance of immersive environments, and mental health improvement.

**Figure 4:**
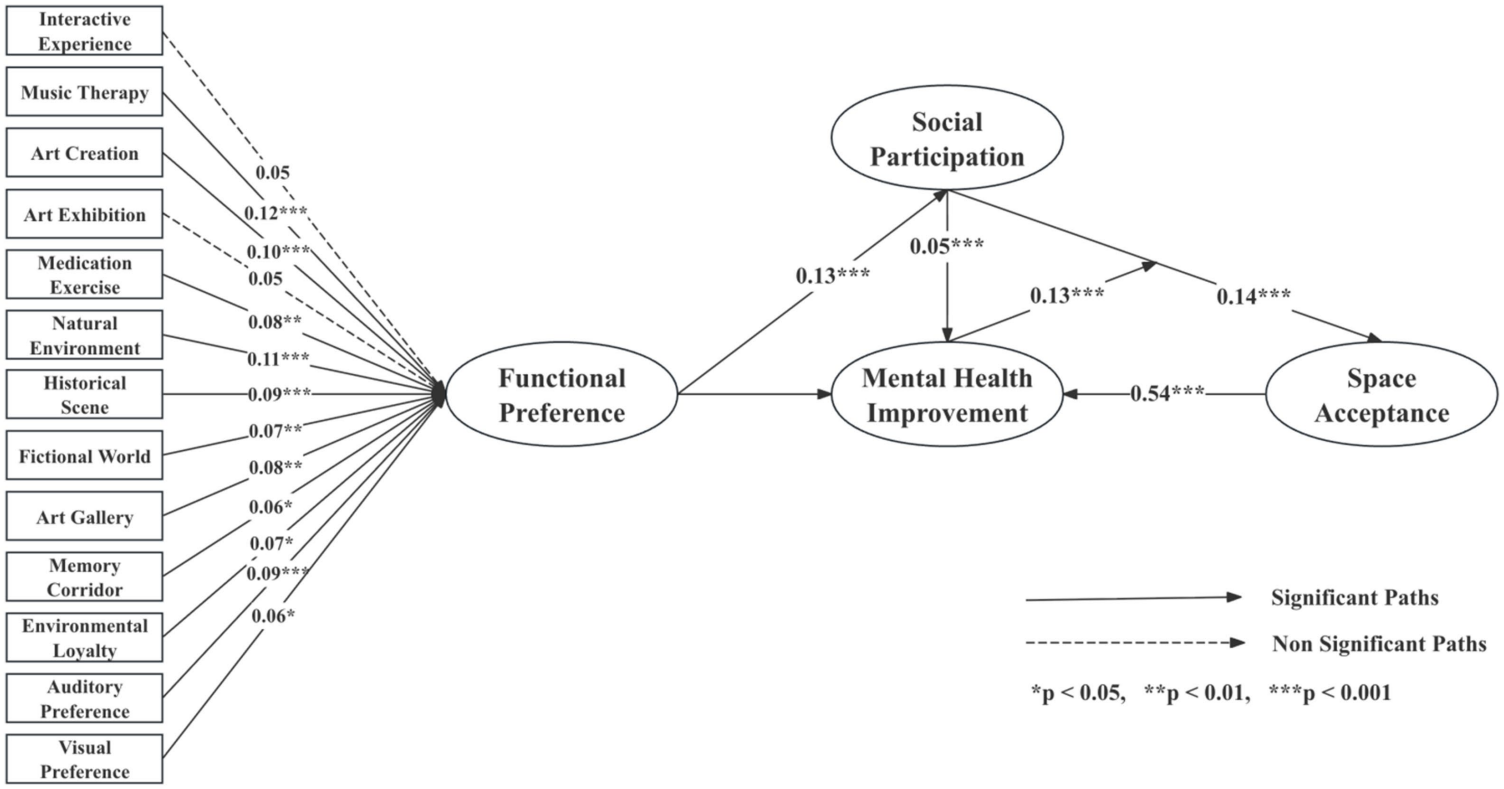
Structural equation model illustrating the complete causal pathways among functional preferences, social participation, mental health improvement, and space acceptance.

The model demonstrated excellent overall fit (*χ*^2^/df = 5.232, RMSEA = 0.047, NFI = 0.982, RFI = 0.975, IFI = 0.985, TLI = 0.980, CFI = 0.985, GFI = 0.978), with all indices meeting or exceeding recommended thresholds. Standardized path coefficients are presented in Table 8, and the structural relationships are illustrated in Figure 4.

**Table 8:** Tests of the relationship between the effects of variables.

Relationship			*b*	*β*	SE	*t*	*p*
Social participation	→	Art healing attitude	0.383	0.545	0.019	19.951	<0.001
Social participation	→	Space acceptance	0.102	0.138	0.022	4.617	<0.001
Social participation	→	Mental health improvement	0.056	0.053	0.025	2.264	0.024
Art healing attitude	→	Space acceptance	0.469	0.448	0.036	12.887	<0.001
Art healing attitude	→	Mental health improvement	0.265	0.177	0.042	6.258	<0.001
Space acceptance	→	Mental health improvement	0.766	0.536	0.041	18.576	<0.001
Functional preference	→	Social participation	0.105	0.908	0.206	5.325	<0.001

Results indicated that functional preferences significantly predicted social participation (*β* = 0.187, *p* < 0.001), suggesting that older adults with stronger interest in specific immersive modules were more likely to engage actively in community activities. Social participation exerted significant positive effects on attitudes toward non-pharmacological interventions (*β* = 0.545, *p* < 0.001), acceptance of immersive spaces (*β* = 0.138, *p* < 0.001), and mental health improvement (*β* = 0.053, *p* = 0.024). Attitudes toward non-pharmacological interventions further predicted acceptance (*β* = 0.448, *p* < 0.001) and directly contributed to mental health improvement (*β* = 0.177, *p* < 0.001). Acceptance of immersive spaces demonstrated the strongest direct effect on mental health improvement (*β* = 0.536, *p* < 0.001).

Mediation analyses confirmed three significant indirect pathways (Table 9). First, social participation indirectly enhanced mental health through attitudes toward non-pharmacological interventions (effect size = 0.101, 95% CI excluding zero). Second, social participation indirectly improved outcomes through acceptance of immersive spaces (effect size = 0.078, 95% CI excluding zero). Third, a chained mediation pathway was identified: social participation improved attitudes toward non-pharmacological interventions, which increased acceptance, and subsequently enhanced mental health improvement (effect size = 0.137, 95% CI excluding zero).

**Table 9:** Model mediation effect test.

Relationship	Effect size	95% lower	95% upper	SE
Social participation → Art healing attitude → Psychological health improvement	0.101	0.063	0.141	0.020
Social participation → Space acceptance → Psychological health improvement	0.078	0.047	0.111	0.017
Social participation → Art healing attitude → Space acceptance → Psychological health improvement	0.137	0.112	0.168	0.014

Taken together, these findings suggest that immersive therapeutic spaces affect psychological outcomes not only directly but also through psychosocial pathways involving functional preferences, attitudes, and acceptance. This highlights the importance of aligning intervention design with older adults’ sensory and activity preferences, as these preferences foster social participation and, in turn, amplify the public health impact of community-based immersive interventions.

## 5 Discussion

### 5.1 Interpretation of key finding

The structural models demonstrate that acceptance of immersive therapeutic spaces is a strong and consistent predictor of mental health improvement among older adults. In the full pathway model, the standardized coefficient for this relationship was 0.536 (Table 8), while hierarchical regression analyses showed similar effects across specifications (Table 4). These findings highlight acceptance as a proximal determinant of psychological outcomes, which fits within emotion regulation models emphasizing that openness to external resources facilitates adaptive coping. This suggests that multi-sensory engagement captures a meaningful share of variance in mental health outcomes, supporting the notion that immersive environments facilitate emotional regulation and stress recovery in later life (98). Recent studies further confirm that immersive interventions can enhance cognitive performance, reduce anxiety, and improve overall psychological well-being in older populations (101, 102).

H2 posited a mediating role of mental health in the link between social participation and acceptance. The mediation model showed that greater social participation was associated with lower depression, anxiety, and stress (*β* values from −0.324 to −0.399, Table 2), and each mental health dimension negatively predicted acceptance (*β* from −0.167 to −0.191, Table 2). Bootstrap estimates confirmed that all three indirect effects were statistically significant with confidence intervals excluding zero (Table 3). This aligns with social support theory, which posits that social ties buffer distress and thereby increase openness to novel interventions (103, 104), and further resonates with emotion regulation frameworks where reduced symptoms free cognitive resources for adaptive engagement.

Additionally, H3 addressed moderation by social participation and baseline mental health. The positive interaction between social participation and acceptance (*b* = 0.291, *p* < 0.001, Table 4) indicates that the association between acceptance and improvement is strongest among those who are more socially active (105). In contrast, negative interactions were observed for depression, anxiety, and stress (*b* from −0.355 to −0.404, all *p* < 0.001, Table 4), and simple slope analyses showed attenuated gains from acceptance at higher baseline symptom levels (Figure 2). Similar trends have been observed in community park studies, where older adults with lower baseline stress derived stronger restorative benefits from multisensory natural environments than those with higher psychological burdens (106). These results suggest that while immersive spaces may be broadly beneficial, their impact is conditioned by the user’s social connectedness and initial psychological burden, which is compatible with stepped-care and tailoring principles in psychosocial intervention design (107, 108).

Beyond these pathways, this study also identified that functional preferences for specific modules, such as music therapy, art creation, or natural environments, were positively associated with social participation. This finding suggests that older adults’ engagement with multisensory spaces is not only a matter of individual psychological benefit but also a driver of collective interaction and community involvement. In public health terms, designing interventions that align with functional preferences may therefore serve as an indirect mechanism to foster social participation, which in turn enhances openness to non-pharmacological interventions and amplifies mental health benefits. Notably, post-hoc analyses indicated that cluster membership was not strongly differentiated by basic socioeconomic indicators such as income, education, or living arrangement, suggesting that the identified preference profiles reflect psychosocial and behavioral orientations rather than structural socioeconomic stratification. This may enhance the applicability of preference-based intervention tailoring across diverse community populations.

### 5.2 Theoretical and practical implications

Theoretically, this study contributes to public health and behavioral sciences by demonstrating that social participation and intervention acceptance jointly form a behavioral pathway to mental health improvement. This pathway can be situated within social support theory, which emphasizes the protective role of social ties in buffering distress and facilitating openness to new interventions, and within emotion regulation frameworks, where multisensory inputs function as external resources that support affective regulation (109). By integrating these perspectives, our findings suggest that community-based immersive environments should be understood not only as technological innovations but also as behavioral health interventions grounded in established theoretical models.

Second, the moderation findings refine expectations about heterogeneity in intervention efficacy. Social embeddedness amplified the benefits of acceptance, whereas high baseline symptoms constrained immediate gains. This dual pattern resonates with stepped-care principles, which highlight the need to stratify intervention intensity based on baseline conditions (107, 108). Consequently, immersive therapeutic spaces may be most effective when implemented in conjunction with tailored support for individuals with higher psychological burden.

Third, the observed module-level heterogeneity indicates that not all immersive elements are equally therapeutic. Activity-oriented modules such as music therapy, art creation, and meditation showed stronger associations with psychological improvement compared to passive or purely visual modules. This is consistent with findings from multisensory stimulation programs in institutional care settings, where activity-based engagement significantly reduced agitation, anxiety, and stress (74, 110). Furthermore, interventions that combined multisensory environments with professional facilitation enhanced life satisfaction and reduced anxiety (101), underscoring the importance of combining design with supportive guidance.

From a practical perspective, these findings highlight the potential of immersive therapeutic spaces to complement evidence-based psychosocial interventions such as CBT, group-based activities, and music therapy. While these remain gold-standard approaches, community-based multisensory environments offer advantages in scalability and accessibility, particularly for non-clinical populations. Importantly, the stronger effects of activity-oriented modules suggest that immersive environments can extend the benefits of existing programs by providing structured, culturally relevant, and technology-supported engagement opportunities. Integrating these approaches into community health promotion may therefore enhance both adherence and long-term effectiveness, offering a feasible pathway for sustainable public mental health interventions.

### 5.3 Limitations and directions for future research

This study has several limitations that should be considered when interpreting the findings. First, the cross-sectional design restricts the ability to infer causality and determine the temporal sequencing of mechanisms, even though advanced modeling techniques were employed. Future longitudinal and experimental studies are required to verify causal pathways and assess the long-term sustainability of intervention effects (107, 108).

Second, the geographic concentration of the sample in selected communities in China constrains the generalizability of the findings to other cultural and regional contexts. As the study was conducted within the Chinese community health context, cultural norms, institutional arrangements, and collective service provision may have influenced participants’ perceptions and acceptance of immersive interventions. Caution is therefore warranted when generalizing these findings to other cultural or healthcare systems. Previous evidence indicates that community-based facilities, environmental features, and cultural norms are closely associated with late-life depression and psychological well-being (111). Although the response rate was exceptionally high due to face-to-face data collection in community settings, the possibility of non-response bias cannot be entirely excluded. Older adults who are socially isolated or less engaged in community activities may have been underrepresented, which may limit the generalizability of the findings. Future research should therefore explore cross-cultural and cross-regional variations in sensory preferences, social participation patterns, and intervention uptake, as cultural differences may influence both acceptance and effectiveness.

Third, this study focused exclusively on community-dwelling, non-clinical populations. While this group represents a critical target for preventive interventions, the findings may not apply to more vulnerable populations, such as residents of long-term care institutions or older adults with clinically significant psychological conditions. Prior studies have demonstrated that multisensory stimulation (MSS/MSE) can reduce agitation and anxiety in residents with dementia (74, 102), and randomized controlled trials combining Snoezelen rooms with therapeutic support have shown positive effects on quality of life (101). Thus, future research should assess the scalability and adaptability of immersive interventions across diverse care settings, ranging from community centers to institutional environments.

Building on these considerations, future research should prioritize three directions: (1) longitudinal and experimental verification of causal mechanisms, (2) examination of cultural and regional variability in sensory and psychosocial responses, and (3) application of immersive interventions across diverse care settings to evaluate scalability, adaptability, and public health relevance.

## 6 Conclusion

This study proposed and empirically tested a conceptual framework linking multisensory interaction design, social participation, and attitudinal factors to psychological well-being among community-dwelling older adults. Using large-scale data and structural equation modeling, the findings confirmed both direct and indirect pathways: higher acceptance of immersive therapeutic spaces was strongly associated with improved mental health, while social participation reduced psychological distress and enhanced receptivity to such interventions.

The results underscore the behavioral and psychosocial mechanisms through which immersive environments support healthy aging. Social participation acted as a protective factor, acceptance of non-pharmacological interventions emerged as a proximal determinant of outcomes, and functional heterogeneity highlighted the stronger therapeutic value of activity-oriented modules such as music therapy, art creation, and meditation. These findings provide theoretical support for social support and emotion regulation frameworks, and offer practical guidance for designing scalable, culturally relevant, and person-centered community programs.

While limited by the cross-sectional design and regional sample, this study contributes important empirical evidence for integrating immersive multisensory environments into public health strategies for aging populations. Future research should prioritize longitudinal and experimental designs to establish causal relationships, explore cross-cultural differences in acceptance and effectiveness, and examine the applicability of immersive therapeutic spaces across diverse community and institutional settings to support their sustainable and equitable implementation.

## Data Availability

The datasets presented in this study can be found in online repositories. The names of the repository/repositories and accession number(s) can be found: https://osf.io/a9f7n/.
