# Supplementary material for: Community-based multisensory environments as preventive public health interventions for mental well-being in older adults: evidence from a large-scale study in China
Source: Front Public Health. 2026 Jan 14;13:1718222. doi: 10.3389/fpubh.2025.1718222 (PMC12847058; doi:10.3389/fpubh.2025.1718222)
Supplement: Supplementary file 1 [file Table_1.docx]

Supplementary Material

# Supplementary Figures and Tables

## Supplementary Tables

**Supplementary Table S1. Demographic characteristics by cluster membership (N = 1,897)**

| **Variable** | **Category** | **Group 1**  **(n = 630)** | **Group 2**  **(n = 703)** | **Group 3**  **(n = 564)** | **χ²** | **p** |
| --- | --- | --- | --- | --- | --- | --- |
| Income (CNY/month) | ≤1000 | 226 (35.9%) | 254 (36.1%) | 205 (36.3%) | 2.32 | 0.888 |
|  | 1001–2000 | 231 (36.7%) | 271 (38.5%) | 223 (39.5%) |  |  |
|  | 2001–5000 | 76 (12.1%) | 83 (11.8%) | 61 (10.8%) |  |  |
|  | ≥5001 | 97 (15.4%) | 95 (13.5%) | 75 (13.3%) |  |  |
| Education level | Primary school or below | 84 (13.3%) | 123 (17.5%) | 78 (13.8%) | 14.86 | 0.137 |
|  | Middle school | 269 (42.7%) | 265 (37.7%) | 239 (42.4%) |  |  |
|  | Vocational/High school | 192 (30.5%) | 238 (33.9%) | 182 (32.3%) |  |  |
|  | Associate degree | 49 (7.8%) | 44 (6.3%) | 47 (8.3%) |  |  |
|  | Bachelor’s degree | 33 (5.2%) | 31 (4.4%) | 16 (2.8%) |  |  |
|  | Master’s degree or above | 3 (0.5%) | 2 (0.3%) | 2 (0.4%) |  |  |
| Living arrangement | Living with children | 306 (48.6%) | 321 (45.7%) | 277 (49.1%) | 4.62 | 0.594 |
|  | Living with spouse | 235 (37.3%) | 290 (41.3%) | 219 (38.8%) |  |  |
|  | Living alone | 47 (7.5%) | 56 (8.0%) | 37 (6.6%) |  |  |
|  | In a nursing home | 42 (6.7%) | 36 (5.1%) | 31 (5.5%) |  |  |

Note:Values are presented as n (% within cluster). Group differences were examined using chi-square tests. Effect sizes (Cramer’s V) were small for all comparisons (income V = 0.025; education V = 0.063; living arrangement V = 0.035), indicating that cluster membership was not primarily driven by basic socioeconomic composition.
